# An Igor Pro 8.01 Procedure to Analyze Pulse Oximetry during Acute Hypoxia Test in Aircrews

**DOI:** 10.3390/s23042327

**Published:** 2023-02-20

**Authors:** Manuel Alvear-Catalán, Claudio Montiglio, Ignacio Perales, Ginés Viscor, Oscar F. Araneda

**Affiliations:** 1 Centro de Medicina Aeroespacial (CMAE), Fuerza Aérea de Chile, Santiago 7550000, Chile; 2 Facultad de Ciencias, Universidad de Chile, Santiago 783090, Chile; 3 Physiology Section, Department of Cell Biology, Physiology and Immunology, Faculty of Biology, Universitat de Barcelona, 08035 Barcelona, Spain; 4 Integrative Laboratory of Biomechanics and Physiology of Effort, (LIBFE), School of Kinesiology, Faculty of Medicine, Universidad de los Andes, Santiago 7620001, Chile

**Keywords:** heart rate, pulse oximetry, software, hypobaric chamber, aircrews, acute hypoxia test

## Abstract

The recognition of hypoxia symptoms is a critical part of physiological training in military aviation. Acute exposure protocols have been designed in hypobaric chambers to train aircrews to recognize hypoxia and quickly take corrective actions. The goal of the acute hypoxia test is to know the time of useful consciousness and the minimal arterial oxygen saturation tolerated. Currently, there is no computer system specifically designed to analyze the physiological variables obtained during the test. This paper reports the development and analytical capabilities of a computational tool specially designed for these purposes. The procedure was designed using the Igor Pro 8.01 language, which processes oxygen saturation and heart rate signals. To accomplish this, three functional boards are displayed. The first allows the loading and processing of the data. The second generates graphs that allow for a rapid visual examination to determine the validity of individual records and calculate slopes on selected segments of the recorded signal. Finally, the third can apply filters to generate data groups for analysis. In addition, this tool makes it possible to propose new study variables that are derived from the raw signals and can be applied simultaneously to large data sets. The program can generate graphs accompanied by basic statistical parameters and heat maps that facilitate data visualization. Moreover, there is a possibility of adding other signals during the test, such as the oxygenation level in vital organs, electrocardiogram, or electroencephalogram, which illustrates the test’s excellent potential for application in aerospace medicine and for helping us develop a better understanding of complex physiological phenomena.

## 1. Introduction

The human brain requires a continuous supply of oxygen to function effectively. It is, therefore, vulnerable to environments with a low availability of this atmospheric gas. At altitude, a decrease in barometric pressure reduces the partial pressure of inspired oxygen, which induces hypoxia in humans [1].

Both civil- and military-crewed airplanes usually operate at altitudes above the physiological barrier of 10,000 feet (3000 m), which implies the need for the aircrew and passengers to use auxiliary systems such as cabin pressurization or a supplemental oxygen supply when using aviation masks [2]. In the event of the failure of these systems, aircrew members or passengers are exposed to the actual flight altitude with the consequence of acute hypoxia exposure, which affects the speed of reaction and ability to carry out more complex actions that involve the use of working memory, short-term memory, and attention [3,4]. The impairment of these functions presents a significant variability among individuals [5], which is relevant since the deficit of cognitive capacities can have consequences on the aircrew’s performance of tasks and expose them to a dynamic environment associated with incidents or accidents during the flight. For this reason, recognizing hypoxia symptoms is a critical component of the training of aircrews [6]. In the 1960s, a program of controlled exposure to hypoxia, generically called “physiological training”, was developed, which is mandatory for the military aircrews of NATO countries and has become standard to be periodically performed (approximately every three to five years), usually being carried out in hypobaric chambers although also under normobaric hypoxia conditions with a reduced oxygen breathing device (ROBD) [7] with different altitude profiles [8].

The most common profiles used in hypobaric chamber training involve exposure at 25,000 feet (7620 m). At this altitude, the heart rate increases as it is stimulated by the sympathetic nervous system, and oxygen saturation decreases to approximately 60% considering the individual variability of each subject [9]. In addition, one of the fundamental objectives of physiological training is to promote the recognition of symptoms associated with hypoxia (hot flashes, paresthesia, dizziness, cognitive deficits, and headache) that may appear in real flight conditions due to hypoxic events (for example, an abrupt depressurization) and, thus, decrease accidents. In addition, during the physiological training test in hypoxia, the presence of these symptoms constitutes the primary alert for the evaluating personnel to administer supplemental oxygen [10].

In addition to symptom monitoring, some hypobaric chambers routinely include the ability to obtain oxygen saturation and heart rate measurements through digital pulse oximetry. Thus, derived from oxygen saturation monitoring, some previous publications have described different segments of the acute hypoxia response curve of potential physiological importance [11,12]. In addition, alternatively to the symptoms, the determination of oxygen hemoglobin saturation is used by some aerospace assessment centers to administer 100% oxygen, with no consensus on the threshold value of this parameter. Thus, some authors point out the administration of supplementary O_2_ from 60% [12], while others used values of 65% [8,13]. Regarding heart rate, this parameter is not habitually included during the test; however, as the study of its response during the test improves, it may be incorporated into monitoring as a helpful tool.

The Chilean Air Force Aeromedicine Center (CMAE) has had a hypobaric chamber since 1981 to physiologically train its aircrews with a periodicity of every three years. All serial data from sensors are transmitted to a concentrator box inside the CMAE M-10 and are transmitted to the first computer, where the trainers can evaluate the aircrew status inside the chamber. Using this system, our center completed 6000 flight hours of physiological training in 2021, allowing us to provide novel information to other centers worldwide that carry out this training. To optimize the physiological analysis of the obtained recordings, we present, in the following report, an innovative computational tool developed as a procedure to be carried out with the Igor Pro 8.01 software that can process, analyze, and present reports and graphs of the results of pulse oximetry recordings obtained during physiological training. The computer code is available in the Supplementary Materials of this article.

## 2. Materials and Methods

**Figure 1:**
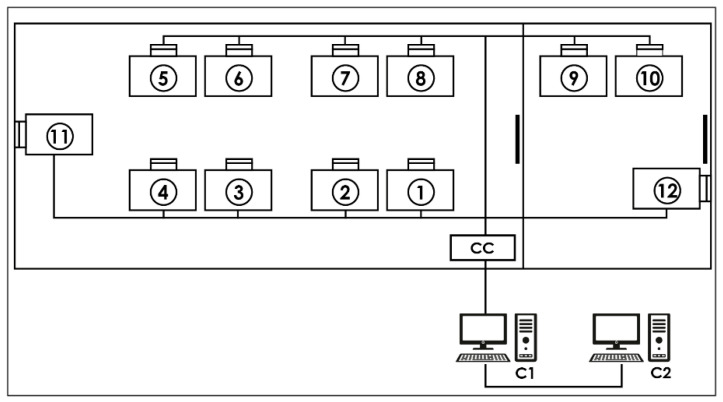
Data acquisition system: Each number represents a place for a crew member equipped with a pulse oximeter. CC—concentrator box that receives the serial data from each location via cables. C1—“MasterBio” computer allowing the storage and display of serial data in real time. C2—“*Analysis*” computer that backs up the serial data.

Acquisition system: The CMAE hypobaric chamber (Environmental Tectonics Corporation ETC, model M-10) is equipped with an integrated physiological acquisition system, denominated “SIMBIO”, that registers the identification number of each activity independently with the date and time, the flight protocol performed, the name of each member of the aircrew trained, and their records of hemoglobin oxygen saturation (reusable sensor with clip installed on the index finger) and heart rate registered through digital pulse sensors (Nonin, Ipod 3211 model). Once the data have been acquired, they are transmitted to a concentrator box located inside the chamber to be sent to the first computer (called “MasterBio”). In this computer, using a computational procedure designed with the Igor Pro 8.01 language (WaveMetrics, Portland, OR, USA), it is possible, in real-time, to observe the data and send them to a second computer (called “Analysis”) for storage and further analysis (for details, see Figure 1).

### 2.1. Igor Pro Procedure Description

To analyze the flights, a procedure called “Physiological Analysis of Hypoxic Simulated Flight” (PAHSF) was developed and designed with Igor Pro 8.01 software (WaveMetrics, Portland, OR, USA). This program is widely used in biomedical research due to its programmability, availability of procedures for specific data analysis purposes, and the generation of high-quality graphics. The software can be obtained, in its free or demo version, from the web page: https://www.wavemetrics.com/products/igorpro (accessed on 16 February 2023). The procedure described in this work is freely available. If a reader wants to try it, we provide the code that works up to the latest version of the program (Igor Pro 9) and an example obtained during a real hypoxia training session in the Supplementary Materials. In addition, to help readers better understand, we have deposited a video in the Supplementary Materials section where the structure and functionalities of the program described in this text are explained.

#### Execution of the Procedure

**Figure 2:**
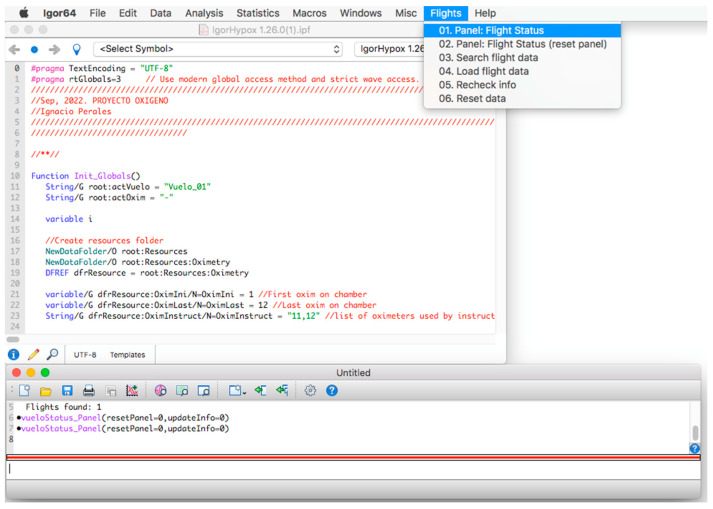
Initial screen of the procedure.

**Figure 3:**
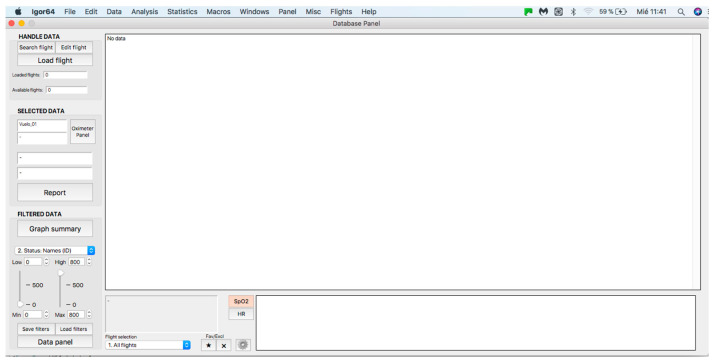
General panel of the database. Handle data—data loading and processing panel (see at the top). Selected data—panel for visualization and analysis of individual curves (see in the middle). Filtered data—filter application panel (see at the bottom).

Once the program is installed, to start its operation, click on the menu bar. Then, on the “Flights” tab and within its options, click on the option “01. Panel: Flight Status” (see Figure 2) and a window called “database panel” will be displayed, as shown in Figure 3.

### 2.2. Handle Data Panel

**Figure 4:**
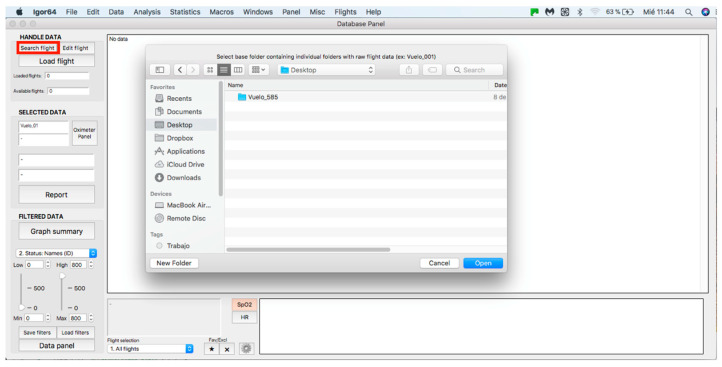
Flight search in the database.

**Figure 5:**
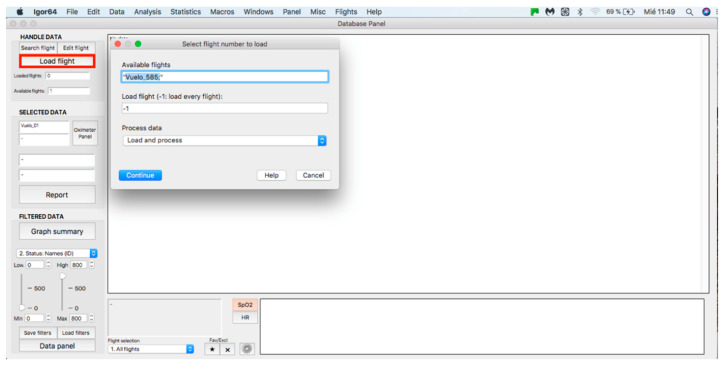
Flight selection from the database.

**Figure 6:**
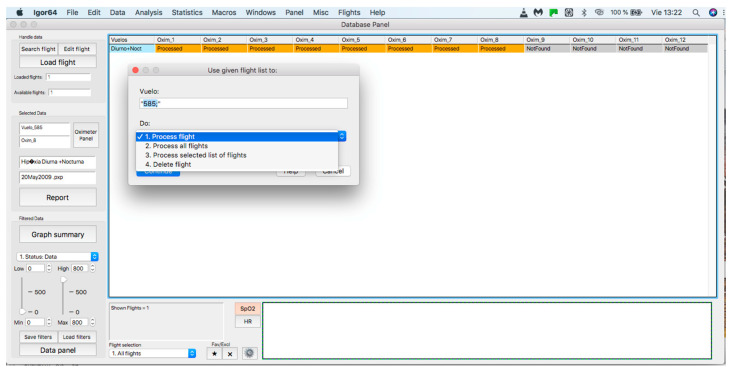
Editing a loaded and processed flight.

**Figure 7:**
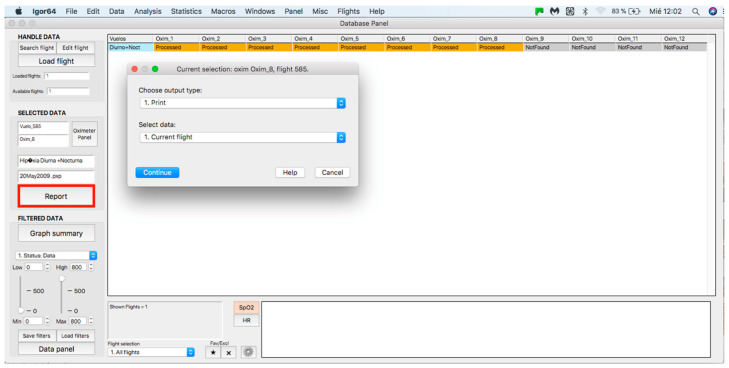
Panel to generate a printed report.

This panel allows for inputting the data associated with the flights to the procedure for further analysis, where data can be either from an individual flight or a sample of several flights. The process starts by clicking on the “Search flight” icon, which displays a window to add flights (see Figure 4 and Figure 5). Once the flight has been added, click on the lower icon, “Load flight” (see Figure 5). There is also the option to edit the selected sample by deleting flights using the function associated with activating the “Edit flight” key (see Figure 6). Finally, it is possible to obtain a report that includes the arterial oxygen saturation and heart rate of a selected subject by clicking the “Report” icon (see Figure 7).

### 2.3. Selected Data Panel

**Figure 8:**
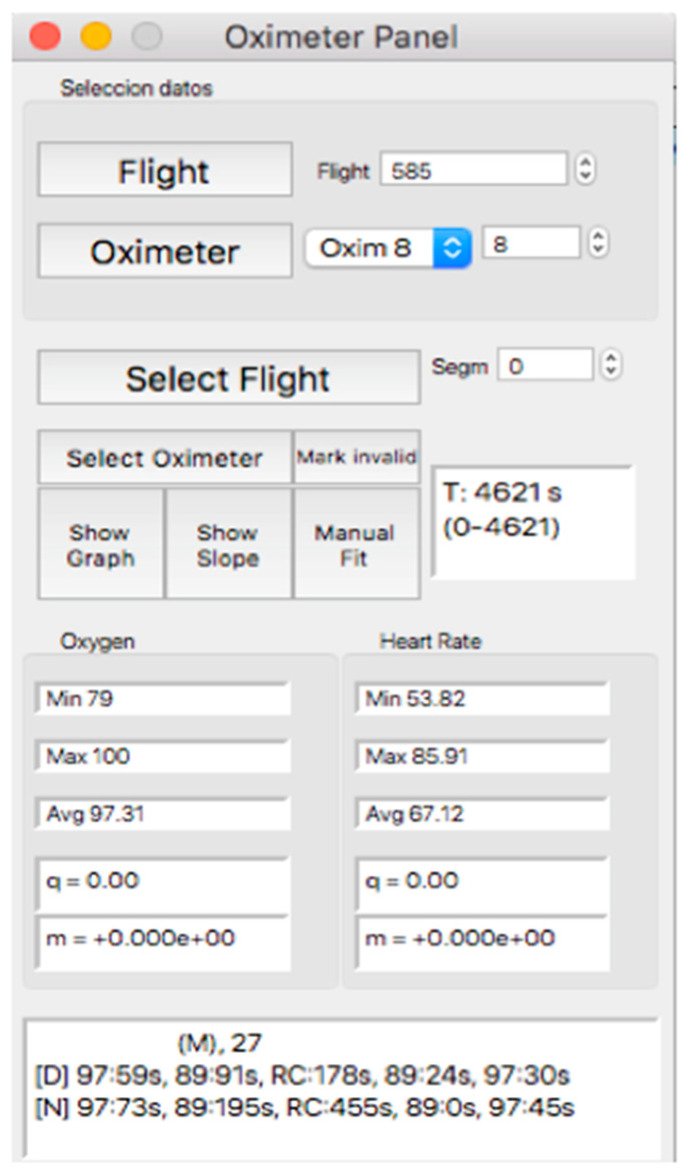
Visual aspect of the subpanel “Oximeter Panel”.

This panel allows for visually inspecting the individual curves, a crucial step for the inclusion of the recording in a later analysis, which consists of determining the continuity of the recording and the coincidence between the oxygen mask connection/reconnection times and the oxygen saturation response. Thus, after selecting the “Oximeter panel” button, a new window appears, the appearance of which is shown in Figure 8.

**Figure 9:**
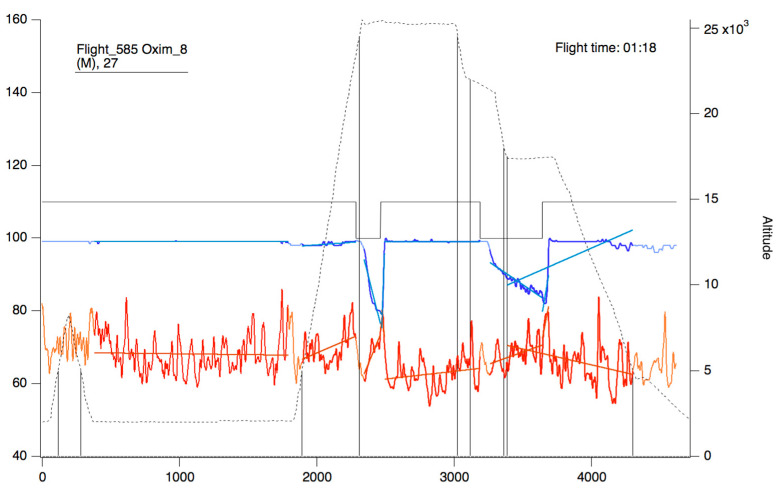
Oxygen saturation curve (blue/sky blue) and heart rate (red/orange). The continuous black line represents the state of connection/reconnection to oxygen. The dashed line represents the flight profile. The lines that appear as a weaker color (light blue/orange) represent the slopes that were calculated for the area of the curve that presents as a more intense color (blue/red).

Within this panel, after selecting the “oximeter” icon, it is possible to visualize an individual oxygen saturation and heart rate curve (see Figure 9).

**Figure 10:**
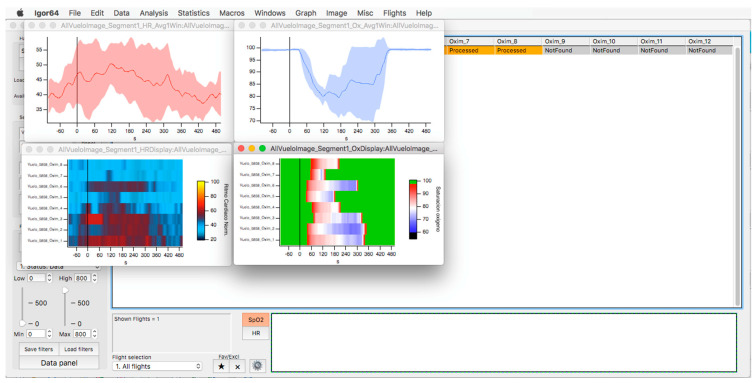
Oxygen saturation (light blue) and heart rate (red) curves are represented as the average and standard deviation of the total number of members of the selected flight (upper graphs). The respective heat maps are shown at the bottom.

In the upper part of the “oximeter panel”, there is a button called “flight”, which adds the corresponding altitude to the selected record (see Figure 9). In addition, the “segm” selector will automatically add the estimated slopes of the different timeline segments (see Figure 9), with the possibility of being corrected manually using the “manual fit” button (see Figure 8), which was incorporated to eventually constitute a criterion for including a subject in a later sample analysis. At the bottom of the panel, four boxes provide the minimum, maximum, average, and slope values for the arterial oxygen saturation and heart rate of the whole recording or selected segments. In addition, the oxygen saturation and heart rate are represented as heat maps by clicking on the “show graph” button, and as graphical representations of the mean and standard deviation or median and interquartile range of all subjects of the flight by clicking on the “show slope” button (see Figure 10). These same graphs and analyses can be obtained from panel three by clicking on the “graph summary” button for a sample of selected flights.

### 2.4. Filter Application Panel

**Figure 11:**
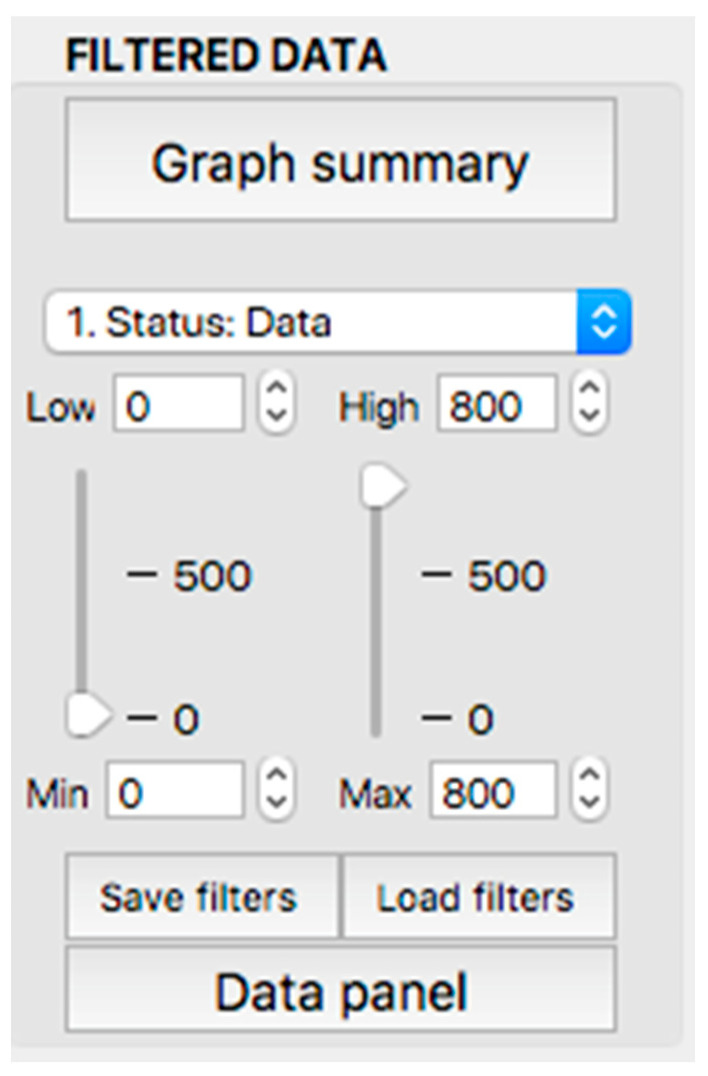
Display of the “filtered data” panel (panel 3).

**Figure 12:**
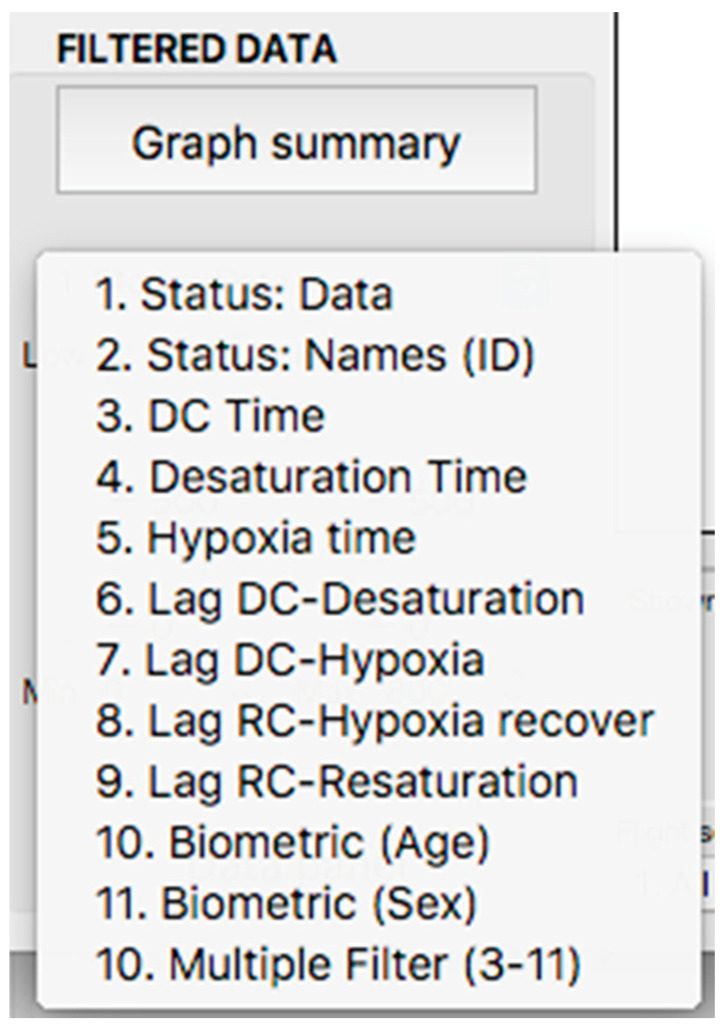
Display of the variable selector subpanel.

**Figure 13:**
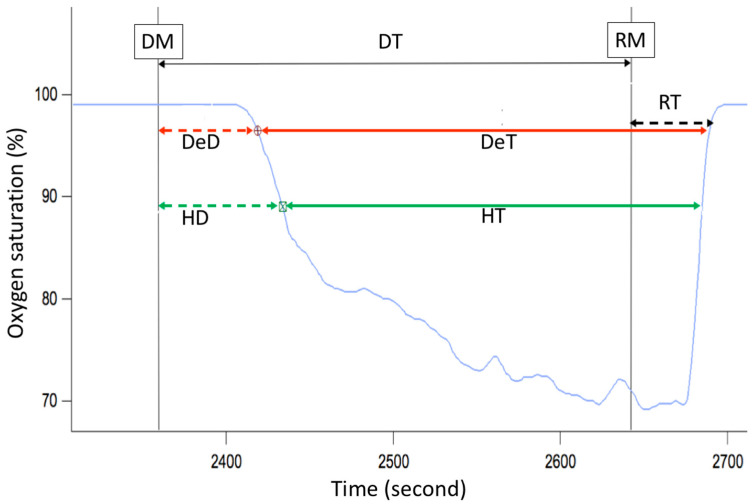
Phases of the oxygen saturation curve (blue line) during a simulated flight. DT—disconnection time (black arrow), DeD—desaturation delay (dashed red arrow), HD—hypoxia delay (dashed green arrow), DeT—desaturation time (solid red arrow), HT—hypoxia time (solid green arrow), RT—recovery time (dashed black arrow), DM—disconnection from oxygen mask, RM—reconnection to oxygen mask.

**Figure 14:**
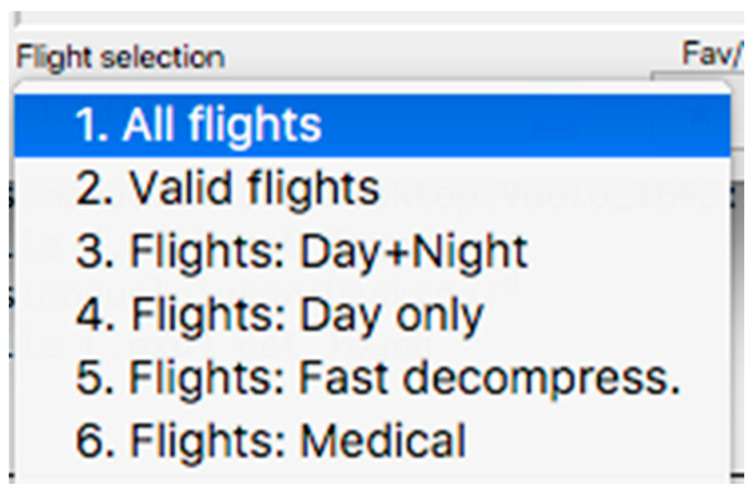
Visualization of the displayed panel with the types of flights performed in the hypobaric chamber.

**Figure 15:**
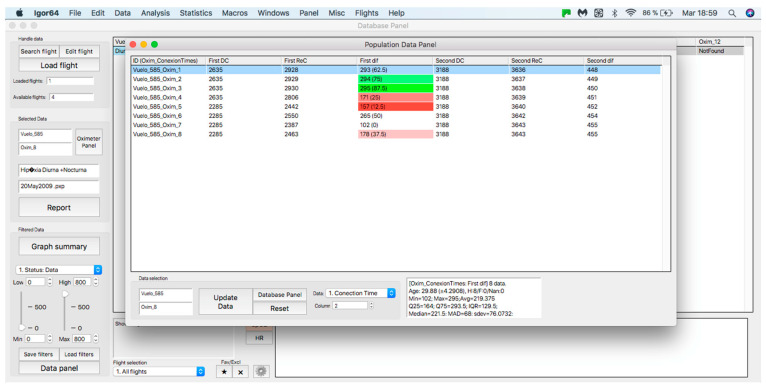
Display of the “sample data panel”.

**Figure 16:**
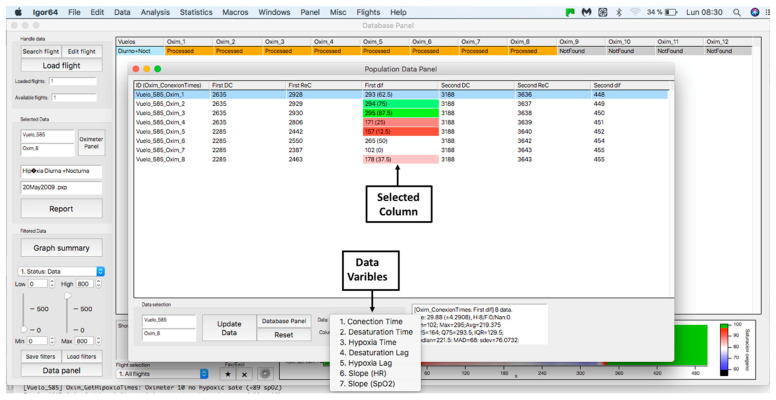
Display of “data” subpanel and selected column.

This panel has the function of delimiting the study sample, for which it has a button called “filter selector” “status: data” (see Figure 11), which, when clicked, displays a subpanel that allows selecting the variables that characterize the subjects (identity, age, and sex), their state of connection/disconnection to the oxygen mask, and finally, the oxygen saturation curve. In the latter, we arbitrarily defined parameters of potential physiological importance for studying the curve. The first was the disconnection phase (DT), which corresponds to the time between disconnection and reconnection to the oxygen mask. The second phase was called desaturation delay (DeD), which corresponds to the time it takes for the oxygen saturation to decrease below 97% after disconnection from the oxygen mask. The third phase was hypoxia delay (HD), which corresponds to the time it takes for the oxygen saturation to decrease below 90% after disconnection from the oxygen mask. The fourth phase was called desaturation (DeT), which corresponds to the time in which the subjects are under 97% oxygen saturation during the test. The fifth phase was defined as the hypoxia phase (HT), which corresponds to the time in which the subjects have an oxygen saturation below 90%, and the last phase corresponded to the recovery phase (RT), which is the time elapsed from the reconnection of the mask until an oxygen saturation of 97% is reached. Figure 12 shows the distribution of these parameters in the respective selector subpanel, whereas Figure 13 represents the determinations as described on an oxygen saturation record. Once the characteristics to be filtered have been determined, it is possible to select ranges manually by determining the minimum and maximum values for each parameter using the selector present in panel 3 (see Figure 11). Once the sample to be analyzed has been defined, this panel has a “save filters” button, which, when clicked, allows saving it for later analysis. To reload a stored sample, click on the “load filters” button, as shown at the bottom of panel 3 (see Figure 11). At the bottom left side of panel 3, there is the “flight selection” selector (see Figure 3), which, when clicked, displays a subpanel that allows selecting the type of flight (total, valid, day and night, day only, decompression, and medical) to be analyzed (see Figure 14). Finally, clicking on the “sample data panel” button displays a window (see Figure 15), which shows the numerical values of the different parameters calculated and provides a descriptive statistical summary of the group in the lower right part. In addition, this window has a selector called “data” and “column” that allows you to select a particular variable (see Figure 16).

## 3. Discussion

This article presents a procedure programmed with the Igor Pro 8.01 software that is capable of quickly processing and analyzing hemoglobin saturation and heart rate records obtained during acute hypoxia tests performed on aircrews in a hypobaric chamber. To the best of our knowledge, this is the first report of a software tool aiming to subjectively analyze the physiological responses of aircrews during a hypoxia test. Among its functions, it allows access to an individual record processed automatically, determining segments and slopes of the curves presented in Figure 9, as well as the possibility of a manual analysis (correcting the proposed automatic analysis) in order to validate it and later analyze it with other data. In addition, the designed procedure has the ability to apply filters to group records according to the particular characteristics of all crew members evaluated and their possible outcomes on the test, such as the effect of sex, the experience of the pilots, the use of drugs, or if there are any pathologies. Once the groups have been selected, it is possible to analyze the data automatically, obtaining unbiased descriptive statistical data, graphical representations, and heat maps, which can be used in the evaluation, monitoring, and search for new innovations in the training protocols of aircrews under these conditions. In addition, it can be useful for the management of safety during real flights and the generation of reports of scientific interest for aerospace medicine. Despite the above, it is essential to note that hemoglobin saturation, although its key role as monitoring tool during aircrew physiological training, has some limitations. First, the SpO_2_ reading should always be considered an oxygen saturation estimate. For example, if an FDA-cleared pulse oximeter reads 90%, the blood’s accurate oxygen saturation is generally between 86–94%. Pulse oximeter accuracy is highest at 90–100% saturation, intermediate at 80–90%, and lowest below 80%. Due to accuracy limitations at the individual level, SpO_2_ provides more utility for trends over time instead of absolute thresholds [14]. To this, we must add inherent factors to the measurement; sensor position and placement, skin color, vasomotion secondary to temperature [15], and problems inherent to the operation of the meters, such as the speed with which they capture variations in saturation [12,16], which becomes important in the segment of the curve where changes in blood pO_2_ generate abrupt variations in saturation [15]. Another relevant aspect is that most sensors are validated to work in clinical environments, with a saturation range between 70–100% [15,16], and the FDA only reviews the accuracy of prescription use oximeters, not OTC oximeters meant for general wellness or sporting/aviation purposes [14]. This has alerted several authors who point out that this parameter should be interpreted with caution in hypoxia conditions [12,15,17]. However, there is an accelerated improvement in sensor technology, nowadays allowing some of them to perform better under acute hypoxia conditions [16].

Our platform also offers the possibility of adding other synchronized physiological inputs such as temperature sensors, NIRS based peripheral tissue oxygenation, and eye movements, which creates opportunities to improve our ability to understand the complexity of the human response to acute hypoxia. Finally, this analysis tool can be used in other areas of science where hypoxia responses are of critical interest, such as high-altitude physiology or respiratory and intensive care medicine.

## Data Availability

The procedure and an example data were uploaded on the Supplementary Material section.

## Supplementary Materials

The following supporting information can be downloaded at: https://www.mdpi.com/article/10.3390/s23042327/s1, Video S1: Tutorial; Computational procedure S2: IgorHypox 1.26.0(1); Experimental data example S3: 21May2022.pxp.
